# Exploration and mortification: Fragile infrastructures, imperial narratives, and the self-sufficiency of British naval “discovery” vessels, 1760–1815

**DOI:** 10.1177/0073275320970042

**Published:** 2020-11-06

**Authors:** Sara Caputo

**Affiliations:** Magdalene College, University of Cambridge, UK

**Keywords:** Royal Navy, exploration, cartography, voyage repairs, warships, empire, Matthew Flinders, Pacific Ocean

## Abstract

Eighteenth-century naval ships were impressive infrastructures, but subjected to extraordinary strain. To assist with their “voyage repairs,” the Royal Navy gradually established numerous overseas bases, displaying the power, reach, and ruthless logistical efficiency of the British state. This article, however, is concerned with what happened where no such bases (yet) existed, in parts of the world falling in between areas of direct British administration, control, or influence. The specific restrictions imposed by technology and infrastructures have been studied by historians interested in naval strategy, but they can also help to reframe national narratives of power or observe the transnational interactions surrounding access to knowledge and resources. This paper discusses the material, cultural, and diplomatic constraints that could appear when vessels, and especially “discovery ships,” sailed in strange waters or sought technical assistance in allied ports. I argue that the “mortification” of some commanders at their vessels’ unfitness for service was an important – and often neglected – element on the palette of emotions undergone by voyagers, capturing their strong sense of ultimate material powerlessness. Such frustration even became embedded in imperial cartography, as shown by the case study of Matthew Flinders. This perspective highlights the limits of naval technology, complicating imperialistic “success stories” and better reintegrating the navy into the history of maritime travel and transportation, from which it is often singled out.

## Introduction

**Figure 1.**
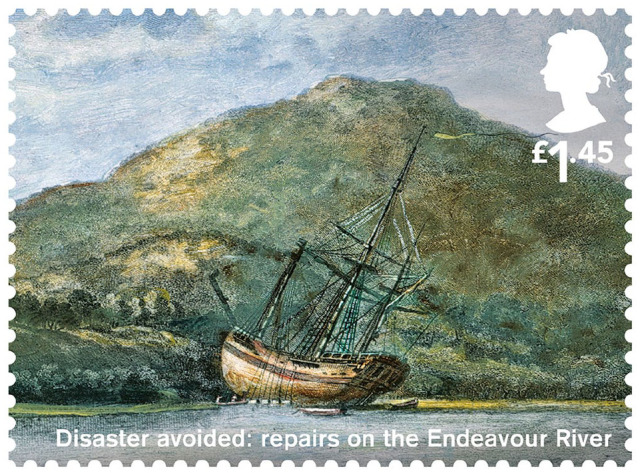
“Disaster avoided: repairs on the Endeavour River.” © Stamp Design Royal Mail Group Ltd (2018). Image source: *Collect GB Stamps* <www.collectgbstamps.co.uk/explore/issues/?issue=22788#collectgbstamps-10>.

**Figure 2.**
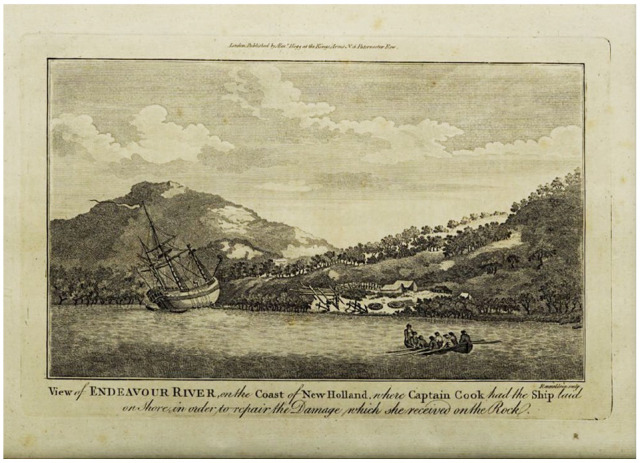
“View of Endeavour River, on the Coast of New Holland, where Captain Cook had the Ship laid on Shore.” Image cropped from: George William Anderson, *A New, Authentic, and Complete Collection of Voyages round the World, Undertaken and Performed by Royal Authority* (London: A. Hogg, 1800), plate ante p.65. Available as part of the Wellcome Collection, under Public Domain Mark: <https://wellcomecollection.org/works/mcsjmyz8>.

**Figure 3.**
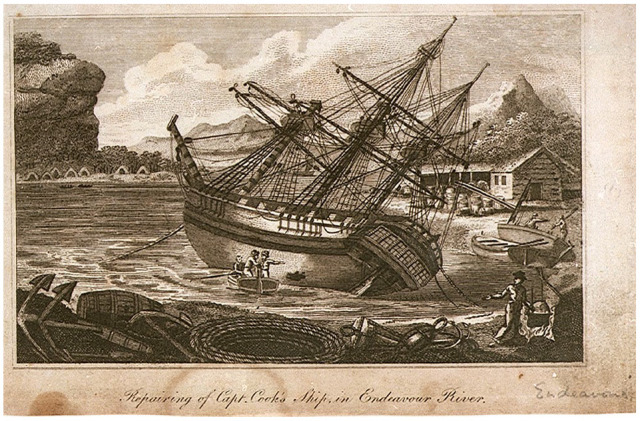
“Repairing of Capt. Cook’s Ship in Endeavour River (Cook’s First Voyage)” (etching, c.1780), PAD5990. © National Maritime Museum, Greenwich, London. <https://collections.rmg.co.uk/collections/objects/110141.html>.

In August 2018, to commemorate the 250th anniversary of James Cook’s first Pacific voyage, the British Royal Mail released a set of ten special stamps, remembering the achievements of the expedition. Next to charts, expedition members, navigational instruments, scientific discoveries, a Tahitian mourner, and a Maori chief, the collection includes one stamp that shows Cook’s vessel careened with makeshift arrangements in Waalumbaal Birri, in the summer of 1770, after it had struck a coral reef; the caption reads: “Disaster avoided: repairs on the Endeavour River” (Figure 1).^1^ The image comes from an engraving that appeared as a plate in the earliest published accounts of the journey (Figure 2).^2^ Other contemporary versions exist, conveying the same impression of activity and technological mastery in a wild and rugged landscape: ship stores are arranged all over the beach, tents and even a cabin have been erected from scratch, and some men carefully assess the leak under the hulk of the beached vessel (Figure 3). The ingenuity and resourcefulness of British navigators, allowing competent ship repairs even in remote corners of the world, have always been part and parcel of the narrative of successful and self-sufficient imperial expansion and “discovery.” However, by turning what often amounted to material defeat into a tale of technological triumph, there is much that this kind of interpretation leaves out.

Keeping naval ships operational abroad required an immense effort. Crews had to be supplied for and taken care of in remote localities. This was the task of the Sick and Hurt Board, which built hospitals, and of the Victualling Board, Navy Board, and Transport Board; corruption, efficiency, and inefficiencies within these bodies have been the object of recent historiographical debates.^3^ Seamen themselves often had to be recruited overseas if too many of them had died or deserted en route.^4^ However, the most fundamental human, material, and technological problem inherent to the relocation of naval power was tending to the vessels themselves, repairing them, and keeping them afloat in different conditions.

**Figure 4.**
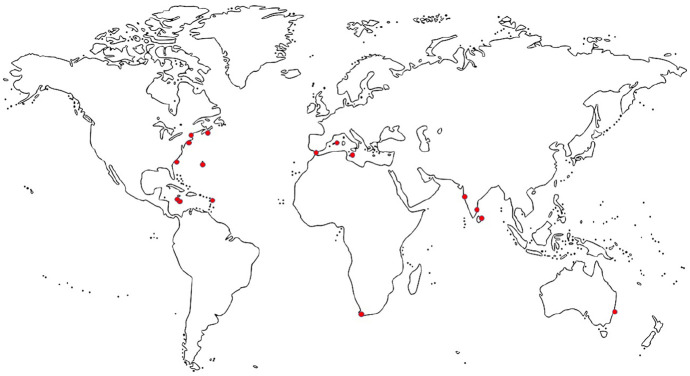
Eighteenth-century British naval bases. These were not all simultaneously under British control, but were created and lost at different times. Blank base map source: <www.needpix.com/photo/81785/world-map-continent-country-geography-planet-earth-africa-asia>.

Eighteenth-century wooden vessels needed regular maintenance and replacement of rotten parts.^5^ In many ways, they were more self-sufficient than modern fuel-powered ships, relying on a constant (if whimsical and fluctuating) source of propulsion, rather than an exhaustible one.^6^ However, intrinsic to their way of traveling were repeated stops for “voyage repairs,” which, unlike today’s “unscheduled ship maintenance,” were more or less an expected feature of long voyages.^7^ The historiography has explored the main solution adopted by the eighteenth-century British Admiralty: the creation of its own overseas bases and stations, supplementing the work of the Royal Dockyards in Britain.^8^ This was an impressive feat, displaying in full the power, reach, and ruthless logistical efficiency of the British fiscal-military state.^9^ Nonetheless, while British naval bases were scattered far and wide, by the end of the century ranging from the West Indies to the Mediterranean, South Africa, the Indian Ocean, and even Australia, significant spaces remained between them, representing weeks or months of sea travel time (see Figure 4). Here we shall examine what happened to naval “discovery” vessels in those parts of the world that fell in between areas of direct British administration, control, or influence.

An extensive “internalist” historiography, primarily focused on the technology itself rather than its context, has meticulously mapped the details and functioning of eighteenth-century naval vessels.^10^ A solid scholarly tradition has also examined the technological and administrative challenges of a naval “blue water” policy, with “lines of communication” stretching across the globe.^11^ However, these studies sought in naval history strategic and political lessons for sea power and warfare. My aim here is, instead, to show how the specific restrictions imposed by naval technology can also be of interest to the social or cultural historian – helping to observe transnational interactions and reframe narratives of national power. What follows, therefore, is partly aligned with Barton C. Hacker’s recent call for a “social history of military technology,” which, less vulnerable to scathing accusations of “antiquarianism” or “utilitarianism,” can appeal even to historians who do not primarily study war itself.^12^ It also echoes John B. Hattendorf’s urging for “specialist” sub-fields in maritime and naval history to overcome their traditional opposition, and explore the options that a joint effort offers.^13^

The difficulty of completing voyage repairs occasionally obstructed military operations. It created situations in which the British fleet suddenly had to rely on external resources. It also affected the imperial scientific explorations sponsored by the navy. Eighteenth- and nineteenth-century “survey sciences” faced unique problems because they intrinsically required mobility and relocation, and had to depend on “reliable action at a distance.”^14^ Historians have examined how the deterioration of both crews’ bodies and technical instruments hampered scientific voyages, but the breakage and decay of the vessels conveying these expeditions have received less attention.^15^ As argued by Richard Sorrenson, ships themselves were in some senses part of the surveyor’s scientific instruments, essential to the task of exploration and shaping its results.^16^ This meant that the very technological infrastructure of the navy, carrying scientific personnel to the other side of the world, could also become one of their main problems: its failure obstructed expeditions in terminal ways, even more than human factors, broken timekeepers, or illness. Looking at these points of insufficiency and failure, in what is in general a story of might and success, highlights some ways in which technology can function as a pivot around which power is not only made – the angle from which the technology-power relationship is usually considered^17^ – but also scrambled. When they have to be transported, some technologies become especially fragile, and this includes technologies of transportation themselves. In this sense, this paper offers a contribution to a growing body of studies stressing the “paradoxical” nature of infrastructures: built for growth, they “deteriorate”; meant to be “solid,” they are in perpetual and intrinsic need to be “retrofitted” and “refurbished”; and they “both mitigate and magnify precarity.”^18^

After a brief overview of some of the material challenges faced by eighteenth-century Royal Navy vessels in general, I discuss how technology, often defective from the start, interfered with the navy’s role as an instrument of imperial exploration and appropriation. Matthew Flinders’s voyage around Australia, finally, offer an example of how the “mortification” of some commanders at their vessels’ unfitness for service even became embedded in imperial cartography.

## Repair challenges and the navy overseas

An eighteenth-century naval ship-of-the-line was one of the biggest and most technologically advanced manufactured objects of its time.^19^ A seventy-four-gun third-rate carried almost thirty miles of line, and was made with timber equivalent to about sixty-seven acres of forest.^20^ By the last wars of the period it measured 170 to 182 feet in length at the gundeck, about forty-seven to forty-nine in width, and had a depth of nineteen to twenty-one feet in the hold.^21^ This is the equivalent of more than twenty train carriages stacked together.^22^ The mainmast towered thirty-six yards above,^23^ like a ten-story building. Even smaller sloops, like those used in journeys of exploration around the world, measured 91 to 113 feet in length and twenty-seven to thirty-five at the beam, a size comparable to that of a Boeing 737-100 tilted to one side.^24^

The size of these vessels ensured that they weathered the open seas, but it multiplied their maintenance needs, especially given that they could be deployed away from Britain for many years at a time. By the final year of any war in the second half of the “long eighteenth century,” a navy ship stationed abroad would have been absent from home for a mean of 641 days (about one year and nine months), a median of 451 (one year and three months), and a staggering maximum of 3253 days (almost nine years).^25^ As for voyages of “exploration,” the mean duration of a round trip was 1009 days, and the median 942.^26^ Of course, time away per se does not mean much, as bases, or even allied ports, were available in many parts of the world. However, they did not always suffice.

All navy ships carried a carpenter, a sailmaker, a boatswain (who took care of the rigging as well as discipline), a caulker, a ropemaker, and various teams working under their orders – “carpenter’s crew,” “sailmaker’s crew,” and so forth.^27^ These specialized artisans were tasked with the small daily repairs, and also intervened when weather, battle, or wear and tear caused more extensive damage, and dockyards were out of range.^28^ They excelled at making the most of broken parts or any locally available resources, yet restoring a vessel to routine operativity was often impossible without spare components. Raw construction materials were frequently imported through a delicate supply network.^29^ Moreover, British metropolitan shipyards were then developing industrial procedures that, some have controversially argued, anticipated the civilian sector by several decades.^30^ The products of such advanced processes could not always be easily replicated in subsidiary facilities. As a result, even in some British bases – let alone away from them – “shortage of stores was the norm, rather than the exception.”^31^

Reliance on the port facilities of allied or neutral powers was also not straightforward. Although eighteenth-century European warships were a common transnational technology, political reasons might prevent mutual support. For example, the Order of St John of Jerusalem, which ruled Malta until 1799, strived for neutrality and non-assistance toward Christian powers at war with each other: from 1768, foreign warships allowed in the harbor of Valletta were capped at four.^32^ Lisbon was fundamental as a repair, refuge, and resupply base for the British fleet throughout the century, but it was abruptly closed if the Portuguese government chose neutrality.^33^ Even where goodwill was not lacking, resources might be. Between 1798 and 1802, the British and Russian fleets were indispensable to the defense of the Kingdoms of Naples and Sicily; as a result, once his arsenals were depleted, the Neapolitan King went as far as supplying the allies with spare parts scavenged from his own warships. These pieces, however, were insufficient and rotten.^34^ In short, even in Europe, reliance on other countries was an intrinsically precarious and uncertain way to operate, and the urgency of repairs could mean depending on “weaker” allies. The material details of these encounters are key to integrating technological naval history into cultural, diplomatic, and transnational history.

## Exploration and mortification

If geographical and technological displacement caused problems to any naval vessel, this was especially true of “discovery ships,” whether warships by build or repurposed merchantmen. Nearly all exploration voyages sponsored by the Royal Navy in the second half of the eighteenth century met with considerable infrastructural issues, forcing the commanders to rely on their crews’ carpentering and manufacturing skills, or on the assistance of authorities in remote settlements of other European powers.

After his expedition in the north-western Pacific, Captain George Vancouver extolled the “friendly and hospitable reception” of the Spanish in California: this included free supplies and, when his own armorer ran away, in January 1793, the very generous loan of the only trained smith in the Monterrey establishment, which solved an otherwise significant material problem. As Vancouver wrote, an armorer “was an artificer of too much importance, to persons in our situation, to be hastily declined.”^35^ During his journey in HMS *Dolphin*, in 1764, Commodore John Byron had to stop at Rio de Janeiro for substantial refits. He hired six Portuguese caulkers, and the officer who later published an account of the expedition was fairly disparaging of their industry and of their country’s resources, but not of their workmanship: “one of our English caulkers would do as much in one day,” he wrote, “as they could in three; but though they are slow and inactive, they perform their work very completely, or else their vessels could not run so many voyages in a shattered condition as they frequently do.” Thousands of miles from the nearest British base, Byron had to make do with what was available, and in fact the episode turned into an opportunity for a technological cultural encounter: the account observed that these men could fix even large seams, making them “as hard as the plank itself, and yet they use not any moisture, as our English caulkers do, except what little proceeds from the constant application of the iron to their mouths.”^36^ Technological observation and “dissemination” are only profitably studied at this sort of micro-level.^37^

In any case, as Dániel Margócsy and Mary Brazelton discuss in the Introduction to this special issue, the relocation of technologies of transportation can result in “conversations” and knowledge exchange between locals and newcomers, but also in “control” issues.^38^ Byron warned “future navigators, particularly those of our own nation,” that the Portuguese at Rio habitually “enticed” or tricked seamen away from the ships that stopped there to refit and resupply; the Portuguese pilot he hired also proved unreliable, and almost wrecked the *Dolphin*.^39^ The most trouble with foreign port authorities was perhaps had by Philip Carteret, as commander of HMS *Swallow*. When he arrived in Celebes in 1767, his ship and his men on their last legs, the Dutch East India Company received him with extreme suspicion and fears of commercial “intrusion,” refused him help at Macassar, and sent him over to Bonthain; the result was that he was easily dragged into local intrigues and convinced of an existing plot to seize his vessel.^40^ Later, in Batavia, after some diplomatic sparring about the previous incident, and complaints against Carteret’s “arrogance,” Dutch carpenters repaired his ship. The *Swallow* was in such bad state that they would have condemned it as unseaworthy, had he not rejected their opinion and taken full responsibility.^41^

In many cases, exploration vessels developed faults as a result of ongoing and exceptional strain. However, the issues were often there from the start. Captain William Robert Broughton of HMS *Providence* remarked in his account that the ship “was singly sheathed with copper,” but he reckoned “it would be proper, that all ships employed in distant voyages should be sheathed with wood, and coppered over the sheathing.”^42^ Without adequate coppering, the hulls of vessels fell prey to the inexorable shipworm *teredo navalis*, which proliferated in tropical latitudes.^43^ For one whole year, the *Providence* made 2–4 inches of water per hour. The leak was eventually repaired only in April 1796, when the ship’s carpenters were able to build a wharf in Nootka and heave down the vessel.^44^ The Vancouver expedition of 1791–5 was in all respects very well equipped, and the *Discovery* was an excellent ship, but even in the initial passage from Spithead to Falmouth the *Chatham*, the armed tender accompanying it, “proved so very crank, as, in some instances, to occasion considerable alarm”: its “crank situation and bad sailing” caused delays and inconveniences throughout, making it lag behind the *Discovery*.^45^

Most notorious of all is the case of Carteret’s HMS *Swallow* during its 1766–9 voyage. The officer complained that it was “an old Vessel . . . and one of the worst, if not the very worst of her kind; in his majesty’s Navy, and was in every respect, but indifferently fitted out.” Even before departure he knew that it was not ready and lacked essentials like a forge: it “was only supply’d with the common, but scanty necessaries, in the manner a Vessell of that kind might be, if bound on an Ordinary voyage, such as to the Mediterranean, & where in case of need, she could be assisted.” As he pointed out, this was inconceivable for “a voyage of discoveries, in the most distant, and dangerous part of the world; where no other helps could be procured, but those we should carry with us.” The situation of the *Swallow* made “common Navigations in Europe, where every Port is open to repair our damage” (as he wrote somewhat hyperbolically) look comparatively safe. However, his requests for improvements had been refused by the Admiralty. Carteret may be a biased commentator, but the conditions of the *Swallow* did mean that the expedition repeatedly teetered on the brink of disaster, and to the end he bemoaned the fact that “such an old Vessell, that was known to have been a bad tool of a ship . . . should be purposely picked for such distant Service, out of all the numerous navy of Great Britain, since there were so many better Ships, lying unemployed, & for want of use, rotting in the harbours of England.”^46^ Exploration vessels were not “neutral” means of transportation, to be taken for granted: they were “tools,” and indeed the most indispensable tool of all. Carteret also alerts us to the contradiction between naval and national might and broken ships, to which we shall return. Much of his rightful indignation was watered down in John Hawkesworth’s Admiralty-sponsored edition of his journal.^47^ This was an attempt to conceal the fact that, oftentimes, explorers were not simply fighting the mighty natural elements and the perils of the unknown, as the heroic narrative goes, but their own incompetently and negligently equipped vessels.

Regardless of anybody’s direct culpability, one common sentiment, almost a trope, recurs throughout voyage accounts, capturing the feelings of commanding officers upon discovering that their ships were defective or damaged. In July 1764, Byron had barely set sail with the *Dolphin* and the *Tamar* when he “had the mortification to find the *Tamar* a very heavy sailer.”^48^ Samuel Wallis set off in August 1766 with the *Dolphin, Swallow*, and *Prince Frederick*, but he “had soon the mortification to find that the *Swallow* was a very bad sailer.”^49^ In August 1776, again scarcely a month after departure, Cook had “the mortification to find our ship [HMS *Resolution*] exceedingly leaky in all her upper works. The hot and sultry weather . . . had opened her seams, which had been badly caulked at first, so wide, that they admitted the rain water through as it fell”: all the crew’s quarters were drenched and made uninhabitable, and the sails “damaged.”^50^ Vancouver had much frustration with masts. In June 1794 he “had the mortification to understand, that just as the carpenters employed on the bowsprit were about leaving off work, they had found it rotten nearly in the middle”; in March the following year, he “had the mortification to learn, that there was not a spar, either at Valparaiso, or in the country within our reach, of a size sufficient to be converted into a [main]mast, for the purpose of replacing our disabled one on board the *Discovery*”; and further “mortification” was caused by discovering that his mainmast was more damaged than he had reckoned, by understanding that the heel of the mast was rotten, even if not sprung like the head, and finally, once the mast was fixed, by the revelation that the mainyard was also largely rotten, and “intirely unfit for service.”^51^ “This was a mortification I did not expect to have met with,” he mourned,

and as there was no possibility of procuring at this place a spar of sufficient size to replace it, the only means we had of repairing the defect was by making a temporary yard out of a spare maintopmast, with the addition of the yard arms of the yard which was decayed, and which I was extremely sorry to observe were by no means in a perfectly sound condition.^52^

This “additional disaster” put an early end to his expedition: “the regret” he “felt in being thus compelled to abandon the examination of this almost unknown, yet interesting part of the coast, is not to be described”; but continuing would have been too dangerous.^53^

Of course, published accounts are written for an audience, and the subtle public rhetoric of blame and self-defense needs to be taken into account. However, the language of mortification seems to capture an important element on the palette of emotions undergone by voyagers, and in particular their strong sense of ultimate material powerlessness.

These feelings are routinely obscured in narratives that only emphasize the overcoming of obstacles and “success story” of naval enterprise in the late eighteenth century. This is illustrated particularly well by one of the most celebrated British expeditions of the time, if we examine it from the point of view of naval technology. Because of its national and imperial significance and mythologization, Matthew Flinders’s mission to chart the Australian coastline is the perfect case study to explore how failing infrastructure can undermine both power dynamics and power narratives.

## Matthew Flinders and his ships

**Figure 5.**
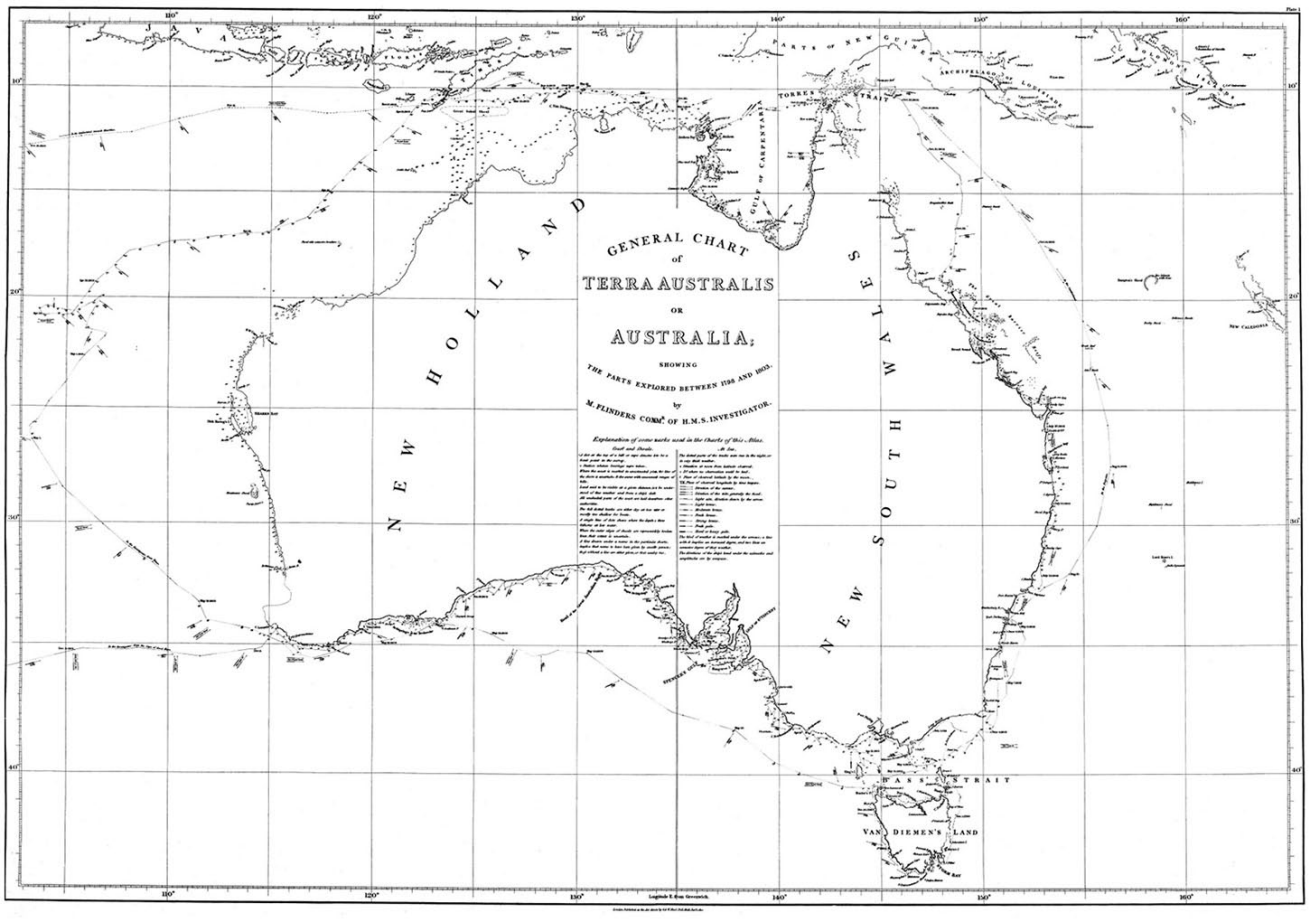
Matthew Flinders, *General Chart of Terra Australis or Australia*. Flinders, *Voyage*, Atlas, Plate I (note 54). Source: “Encounter 1802–2002,” State Library of South Australia, B 1298521 <https://encounter.collections.slsa.sa.gov.au/collection/B12985211_92.htm> [accessed 03/10/2020].

Between 1801 and 1803, by Admiralty orders, the Royal Navy commander and hydrographer Matthew Flinders sailed HM Sloop *Investigator* along the coasts of “New Holland” (present-day Australia), where “His Majesty’s colony of New South Wales” was located, producing detailed charts (Figure 5).^54^ His expedition has been the object of an extensive literature, including biographical and narrative accounts, and work by historians of science and cartography.^55^ Little has been said, however, on what it reveals about the navy and its technology.

Flinders’s sloop was in poor condition and increasingly needing emergency “voyage repairs,” for which the crew could count on no one but themselves. In November 1802, in Torres Strait, it had been leaking up to fourteen inches per hour; at the first suitable anchorage, on Sweers Island, the carpenters discovered two rotten planks, and “patched up the bad part.”^56^ This was not the end of the problems, as during their caulking they came across more and more damage: “report after report,” Flinders wrote, “was brought to me of rotten places found in different parts of the ship, – in the planks, bends, timbers, tree-nails, &c., until it became quite alarming.”^57^ The carpenter and the master concluded that the sloop could not survive a “strong gale,” getting aground, or heaving down for careening: it was given a six-month prognosis in good weather, with the warning that “in twelve months there will scarcely be a sound timber in her.”^58^ Flinders was distraught: his goal had been to achieve the definitive charting of the Australian coastline, even taking the risk of navigation close to shore. “But with a ship incapable of encountering bad weather, – which could not be repaired if sustaining injury from any of the numerous shoals or rocks upon the coast, – which, if constant fine weather could be ensured and all accidents avoided, could not run more than six months; – with such a ship, I knew not how to accomplish the task.”^59^ In March 1803 he abandoned the close survey to return to Port Jackson, a long and troubled journey.^60^ There, on June 14, the master builder and other officers, after a quick examination, decreed that the *Investigator* was “not worth repairing in any country, and that it is impossible in this country to put her in a state fit for going to sea.”^61^ Provisionally patched up, the sloop was subsequently put to use in the colony, and eventually undertook a very precarious return voyage to England in 1805; it was only there that, after being sold into the merchant service, it could undergo proper refitting, and had a further long career, terminating as late as 1872.^62^

As for Flinders, of the handful of ships available in that remote part of the world, none would have been a fit replacement without several months of makeshift, uncertain repairs, so he chose to head back to England aboard one of them, HMS *Porpoise* – which was wrecked on a reef.^63^ Miraculously returned to Port Jackson, he sailed again in the badly leaking HMS *Cumberland*: along the way the small Dutch settlement of Coepang offered no opportunity to repair the pumps, or pitch to seal the seams, so the ship was obliged to call at French-occupied Mauritius. The war with France had just recommenced; Flinders was imprisoned and stranded there for six and a half years.^64^

**Figure 6.**
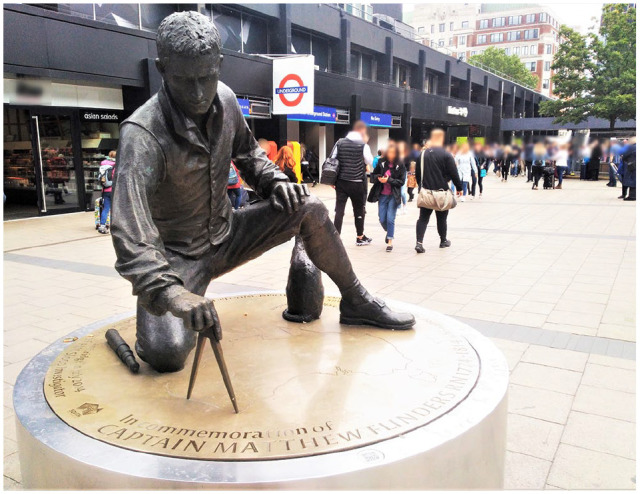
Matthew Flinders Memorial Statue at Euston Station (photograph by Sara Caputo).

Distance from home ports and lack of resources had been the crucial factors provoking the demise of Flinders’s mission. The technological limitations of transporting technologies of transportation to the other side of the globe had both put an early end to the survey, and frustrated any hope of resuming it. Flinders’s struggles to keep afloat and continue with the allotted task – which ultimately did result in carefully made, if incomplete, charts – can be seen as evidence of the professional quality and heroic resourcefulness of the Royal Navy, and of the long reach and stubborn expansionism of the British empire. In this vein they have been celebrated for centuries, in both British and Australian memorialization.^65^ A bronze statue of Flinders, bent over his charts in an elegantly classical pose, in the very act of empire-building, has stood in the middle and later at the entrance of Euston Station, in London, since the bicentenary of his death in July 2014 (Figure 6).^66^ Yet these struggles for survival can also tell a parallel story of weakness, compromise, and disempowerment in the face of natural elements and constrained resources, and nowhere is this more apparent, to a cultural historian of naval technology, than in Flinders’s very charts.

At first sight, these charts, commissioned by the British Admiralty, are themselves an embodiment of the national imperialistic myth. Eighteenth-century maritime science oscillated between cosmopolitan international collaboration and, by 1800, growing nationalisms.^67^ Hydrography in particular had crucial military, economic, and imperial significance, inextricably embedding the surveyors’ work within wider structures of power.^68^ “Accuracy” itself can be a “talisman of authority.”^69^ Whatever the carefulness and apparent scientific objectivity with which they are traced, maps, as a “form of knowledge,” cannot escape the influence of cultural biases and mechanisms of power, as critical cartography has shown.^70^ While Flinders mostly avoided openly patriotic themes in his choice of toponyms for “newly discovered” places, he paid tribute to several British personalities, most notably the three First Lords of the Admiralty, Spencer, St Vincent, and Yorke, who were his patrons (and, in St Vincent’s case, national heroes); friends and shipmates featured, together with labels like “Point Dover” for a white cliff.^71^ He also preferred “Terra Australis,” or “Australia,” as general name for the continent, because it prevented “New South Wales” from becoming part of “New Holland.”^72^ Labeling and measuring a territory, and positioning it in relation to Greenwich, are plain forms of appropriation.^73^ However, paying more attention to the specific technological features embedded in Flinders’s charts reveals a stark counter-narrative.

Like many contemporary explorers and cartographers, Flinders meticulously marked the *Investigator*’s track, with relative dates.^74^ His charts are dynamic, expressing movement next to fixed geographical features, “wayfinding” next to “map-making.”^75^ Choosing to record an individual journey, beside universal and supposedly unchanging features, is in itself an epistemological declaration, a scientist’s way of turning a chart into a falsifiable, provisional, case-specific theory, rather than an impartial statement. Tracks on eighteenth-century charts act as the concrete manifestation of the ship’s role as a surveying instrument.^76^

**Figure 7.**
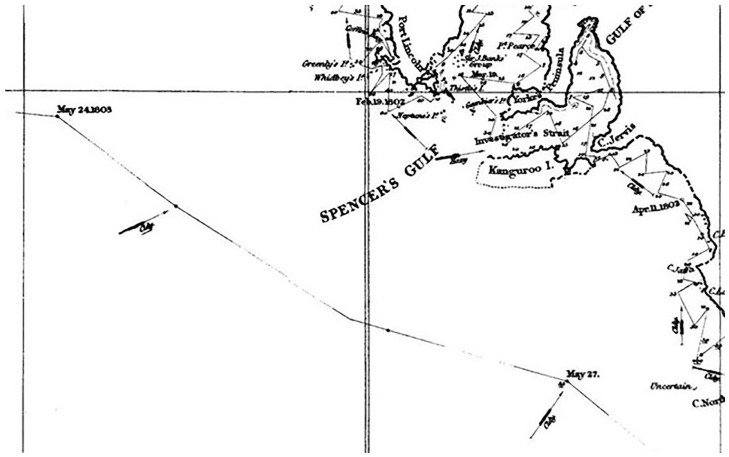
Detail of the route of HMS *Investigator*. From Flinders, *Voyage*, Atlas, Plate I (note 54). Cropped from: “Encounter 1802–2002,” State Library of South Australia, B 1298521 <https://encounter.collections.slsa.sa.gov.au/collection/B12985211_92.htm> [accessed 06/12/2014].

Indeed, this narrative, temporal aspect of the charts makes the ship itself visible and contains a story hidden to a superficial glance. The graphic record allows us to calculate the speed of navigation, seldom reported in Flinders’s published journals. For instance, between May 24 and 27, 1803, just before its arrival in Port Jackson, the *Investigator* covered 395 nautical miles, hence doing an average of 5.49 knots (Figure 7).^77^ This is a respectable speed for a ship of the time, especially “collier-built” and “deeply laden.”^78^ Not necessarily so, however, if sailing with a wind like the one marked on the chart, predominantly a “fresh breeze” (fourth degree on a scale of seven, or a five on the Beaufort scale), coming from one of the best angles – the vessel is “on broad reach,” in technical terms.^79^ The sloop was then rather far from the coast, it had ceased to take any measurements which would have slowed its pace, and in fact Flinders was making all the sail he could to return to Port Jackson as fast as possible, and avoid further casualties among his severely sick crew.^80^ Therefore, we can consider this measurement an accurate gauge of the capabilities of the *Investigator*: it was, by then, making water at the rate of five inches an hour, and would have sunk if the wind had come from either the larboard side or the larboard or starboard quarters, pushing below the waterline the most rotten parts of its hulk.^81^ Similar calculations could of course be performed throughout the *Atlas*, and cast some more light on the British Admiralty’s priorities, resources, and commitment to scientific exploration: Britain possessed the largest fleet on the globe (285 vessels in 1800), and yet – familiar refrain – “no better ship could be spared.”^82^ The truth is that the *Investigator* had started leaking, to Flinders’s “mortification,” as soon as it left England.^83^ Through the slow, limping black track of the sloop, the deficiencies of his patrons are thus engraved into the chart, which consequently tells, next to the grand narrative of British power impressing commuters at Euston, a more subversive story subtly undermining it.

## Conclusion

The eighteenth-century British Navy was unquestionably a successful tool of global power. However, any flat narrative of its achievements, now firmly embedded in national mythology, obscures the limitations of such power. Many of these limitations stemmed directly from the “paradoxical” quality of infrastructures:^84^ the very technology that permitted naval operations in distant seas could, when it ceased to function, become the weakest link in the chain.

Navy ships would be away from British shores for years at a time, regardless of their build or the availability of local resources. Especially when away from British bases, men-of-war were forced to rely on the often precarious, whimsical, or limited help of foreign ports. Royal Navy vessels were not and could not be fully self-sufficient or self-reliant, because complex technologies and infrastructures rarely are. This fact is strikingly embedded in some of the most celebrated feats of naval expansion, hidden beneath the layers of human heroism that have been emphasized instead.

The points where British naval power found itself overstretched, and figuratively and literally “out of its comfort zone,” afford examples of bravery and resourcefulness to those who set out to build national pantheons, important considerations to the naval strategist, but also a different type of insight. On the backdrop of glorious victories and worldwide domination, material fragility, displacement, inadequacy, and dependence also featured, and they help us to tell an alternative, or rather complementary, history of the eighteenth-century British Navy. The interplay between travel distance and the relative brittleness of naval technology can be profitably studied by historians interested in technological transfer, cultural interactions, and transnational contacts. The material difficulties of the sailing navy thus allow us to reintegrate it into maritime history and the history of global travel and transportation, from which it is often implicitly singled out.

## Footnotes

1. Royal Mail, “Captain Cook and Endeavour,” *Collect GB Stamps*, 2018, <www.collectgbstamps.co.uk/explore/issues/?issue=22788> [accessed 20/09/2020]. For Cook’s own account of the episode, see J. C. Beaglehole, *The Journals of Captain James Cook on His Voyages of Discovery: The Voyage of the Endeavour, 1768–1771* (Cambridge: Cambridge University Press, 1968), pp.343–69. The scholarship on the stop at “Endeavour River” has mainly explored the resulting complex encounter with Indigenous people. See e.g. Stuart Murray, “‘Notwithstanding Our Signs to the Contrary’: Textuality and Authority at the Endeavour River, June to August 1771,” in Glyndwr Williams (ed.), *Captain Cook: Explorations and Reassessments* (Woodbridge: The Boydell Press, 2004), pp.59–76.

2. John Hawkesworth, *An Account of the Voyages Undertaken by the Order of His Present Majesty for Making Discoveries in the Southern Hemisphere. . .*, 3 vols (London: W. Strahan and T. Cadell, 1773), Vol. III, Plate 19, by William Byrne, ante p.557 (see also Figure 2).

3. Erica Charters, *Disease, War, and the Imperial State: The Welfare of the British Armed Forces during the Seven Years’ War* (Chicago: The University of Chicago Press, 2014); Roger Knight and Martin Wilcox, *Sustaining the Fleet, 1793–1815: War, the British Navy and the Contractor State* (Woodbridge: The Boydell Press, 2010); Janet Macdonald, *The British Navy’s Victualling Board, 1793–1815: Management Competence and Incompetence* (Woodbridge: The Boydell Press, 2010); James Davey, *The Transformation of British Naval Strategy: Seapower and Supply in Northern Europe, 1808–1812* (Woodbridge: The Boydell Press, 2012).

4. Christian Buchet, “La Royal Navy et les levées d’hommes aux Antilles (1689–1763) : difficultés rencontrées et modalités évolutives,” *Histoire, économie et société* 9:4 (1990): 521–43; Sara Caputo, “Towards a Transnational History of the Eighteenth-Century British Navy,” *Annales Historiques de la Révolution Française* 397 (2019): 13–32.

5. Jonathan G. Coad, *The Royal Dockyards 1690–1850: Architecture and Engineering Works of the Sailing Navy* (Aldershot and Brookfield, VT: Scolar Press, 1989), p.305.

6. Ibid.

7. U.S. Government Accountability Office, *Defense Logistics: Navy Needs to Develop and Implement a Plan to Ensure that Voyage Repairs are Available to Ships Operating Near Guam When Needed* (May 12, 2008) <www.gao.gov/products/A82096> [accessed 23/02/2019].

8. Coad, *Royal Dockyards*, pp.305–77 (note 5); Daniel A. Baugh, *British Naval Administration in the Age of Walpole* (Princeton, NJ: Princeton University Press, 1965), pp.341–72; Duncan Crewe, *Yellow Jack and the Worm: British Naval Administration in the West Indies, 1739– 1748* (Liverpool: Liverpool University Press, 1993); Malcolm Lester, “Vice-Admiral George Murray and the Origins of the Bermuda Naval Base, 1794–96,” *The Mariner’s Mirror* 94:3 (2008): 285–97. On the British dockyards see also: Roger Morriss, *The Royal Dockyards during the Revolutionary and Napoleonic Wars* (Leicester: Leicester University Press, 1983); Philip MacDougall, *Royal Dockyards* (Newton Abbot: David & Charles, 1982); Kenneth Lunn and Ann Day (eds.), History of Work and Labour Relations in the Royal Dockyards (London and New York: Mansell, 1999); J. M. Haas, A Management Odyssey: The Royal Dockyards, 1714–1914 (Lanham, MD: University Press of America, 1994), pp.1–65.

9. Jonathan Coad, “To Serve the Fleet in Distant Waters. Buildings of the Georgian Royal Navy’s Overseas Bases,” *Transactions of the Naval Dockyards Society* 5 (2009): 51–63, 62.

10. A prominent journal in the field has traditionally been *The Mariner’s Mirror*. See also, e.g.: Brian Lavery, *The Ship of the Line – Volume I: The Development of the Battlefleet 1650–1850* (London: Conway Maritime Press, 1983); Robert Gardiner, *Frigates of the Napoleonic Wars* (London: Chatham Publishing, 2000); John Harland, *Seamanship in the Age of Sail: An Account of the Shiphandling of the Sailing Man-of-War 1600–1860, Based on Contemporary Sources* (London: Conway, 1984). For my use of the term “internalist” see John M. Staudenmaier, “Recent Trends in the History of Technology,” *The American Historical Review* 95:3 (1990): 715–25, 716–17.

11. A. T. Mahan, *Naval Strategy Compared and Contrasted with the Principles and Practice of Military Operations on Land: Lectures Delivered at U. S. Naval War College, Newport, R. I., between the Years 1887 and 1911* (Boston, MA: Little, Brown, and Company, 1911); N. A. M. Rodger, “Weather, Geography and Naval Power in the Age of Sail,” *The Journal of Strategic Studies* 22 (1999): 178–200; Jeremy Black and Philip Woodfine (eds.), *The British Navy and the Use of Naval Power in the Eighteenth Century* (Leicester: Leicester University Press, 1988); John B. Hattendorf, *Naval History and Maritime Strategy: Collected Essays* (Malabar, FL: Krieger Publishing Company, 2000).

12. Barton C. Hacker, “The Symposium on the Social History of Military Technology: ICOHTEC 2006–2013,” *Icon* 20:1 (2014): 74–83; Barton C. Hacker, “Introduction: Why the Social History of Military Technology,” *Icon* 14 (2008): 1–2.

13. Hattendorf, *Naval History*, pp.267–74 (note 11).

14. Simon Naylor and Simon Schaffer, “Nineteenth-Century Survey Sciences: Enterprises, Expeditions and Exhibitions,” *Notes and Records: The Royal Society Journal of the History of Science* 73:2 (2019): 135–47, esp. 139–40.

15. See e.g. on health issues: Glyn Williams, “Scurvy on the Pacific Voyages in the Age of Cook,” *Journal for Maritime Research* 15:1 (2013): 37–45; Christopher Lawrence, “Disciplining Disease: Scurvy, the Navy, and Imperial Expansion, 1750–1825,” in David Philip Miller and Peter Hanns Reill (eds.), *Visions of Empire: Voyages, Botany, and Representations of Nature* (Cambridge: Cambridge University Press, 1996), pp.80–106. On instrument fragility: Alexi Baker, “‘Precision’, ‘Perfection’, and the Reality of British Scientific Instruments on the Move During the 18th Century,” *Material Culture Review* 74–75 (2012): 14–29; Charles W. J. Withers, “Geography and ‘Thing Knowledge’: Instrument Epistemology, Failure, and Narratives of 19^th^-Century Exploration,” *Transactions of the Institute of British Geographers* 44 (2019): 676–91; Simon Schaffer, “Easily Cracked: Scientific Instruments in States of Disrepair,” *Isis* 102:4 (2011): 706–17, 709–11; Eóin Phillips, “Instrumenting Order: Longitude, Seamen and Astronomers, 1770–1805,” in Fraser MacDonald and Charles W. J. Withers (eds.), *Geography, Technology and Instruments of Exploration* (London: Routledge, 2015), pp.37–55, 45–6.

16. Richard Sorrenson, “The Ship as a Scientific Instrument in the Eighteenth Century,” *Osiris* 11 (1996): 221–36.

17. See e.g. Daniel Sarewitz, “Technology and Power,” in Stig Andur Pedersen et al. (eds.), *A Companion to the Philosophy of Technology* (Chichester: Wiley-Blackwell, 2009), pp.344–6.

18. Cymene Howe et al., “Paradoxical Infrastructures: Ruins, Retrofit, and Risk,” *Science, Technology, & Human Values* 41:3 (2016): 547–65.

19. John A. Tilley, *The British Navy and the American Revolution* (Columbia, SC: University of South Carolina Press, 1987), p.xv.

20. MacDougall, *Royal Dockyards*, pp.13, 97 (note 8).

21. Lavery, *Ship of the Line*, pp.184–5, 208 (note 10).

22. A Class 466 British regional train carriage, for instance, measures 20.80m×2.80m (and about four meters in height): Angel Trains, “Class 466 – South Eastern,” <www.angeltrains.co.uk/Products-Services/Regional-Passenger-Trains/24> [accessed 29/04/2019].

23. Lavery, *Ship of the Line*, p.208 (note 10).

24. Randolph Cock, “Precursors of Cook: The Voyages of the *Dolphin*, 1764–8,” *The Mariner’s Mirror* 85:1 (1999): 30–52, 47; Rif Winfield, *British Warships in the Age of Sail, 1714–1792: Design, Construction, Careers and Fates* (Barnsley: Seaforth Publishing, 2007), p.1826; Peter Goodwin, *Nelson’s Arctic Voyage: The Royal Navy’s First Polar Expedition 1773* (London: Adlard Coles, 2019), p.42; Boeing Commercial Airplanes, *737 Airplane Characteristics for Airport Planning* (2013), pp.28, 42 <www.boeing.com/assets/pdf/commercial/airports/acaps/737.pdf> [accessed 02/07/2020].

25. I collected this data using the Admiralty Ship List Books for February 1763, September 1783, March 1802 (the final months of the first three conflicts, respectively), and July 1813 (the last month of the Napoleonic Wars for which List Books survive): The National Archives, Kew, ADM 8/39, List Book, 1763; ADM 8/59, List Book, 1783; ADM 8/83, List Book, Jan.–Jun. 1802; ADM 8/100, List Book, 1812–13. The records report the situation on the first day of each month. The stations covered are, varying slightly from war to war, Newfoundland, North America, Portugal, the Leeward Islands, Jamaica, South America, the Mediterranean, the Cape of Good Hope, and the East Indies, plus “Convoys and Cruizers” on general service. Excluded from the sample are vessels stationed in British or Irish waters or deployed in squadrons that bore no sailing date, as well as 285 others with missing, ambiguous, or clearly wrong dates (e.g. subsequent to the day of reckoning), or marked as built or commissioned overseas. The final database contained 572 vessels of all rates and types. When only the month and year of departure were given (1802 sample, occasionally), I uniformly picked the fifteenth day.

26. This data refers to a total of thirteen completed voyages between 1764 and 1795 (when vessels sailed together on an expedition I have counted each as a distinct journey only if they became separated). See the Appendix for details.

27. Michael Lewis, *A Social History of the Navy 1793–1815*, new ed. (London and Mechanicsburg, PA: 2004), pp.261–6.

28. Coad, *Royal Dockyards*, p.305 (note 5).

29. James Davey, “Securing the Sinews of Sea Power: British Intervention in the Baltic 1780–1815,” *The International History Review* 33:2 (2011): 161–84.

30. Carolyn C. Cooper, “The Portsmouth System of Manufacture,” *Technology and Culture* 25:2 (1984): 182–225.

31. Crewe, *Yellow Jack*, pp.263–84, 271 (note 8).

32. Desmond Gregory, *Malta, Britain, and the European Powers, 1793–1815* (Cranbury, NJ: Associated University Presses, 1996), pp.40–1.

33. Baugh, *British Naval Administration*, p.342 (note 8); Martin Robson, *Britain, Portugal and South America in the Napoleonic Wars: Alliances and Diplomacy in Economic Maritime Conflict* (London and New York: I. B. Tauris, 2011), pp.36–40, 147–51, 162–95.

34. Sara Caputo, “Foreign Seamen and the British Navy, 1793–1815” (PhD thesis, University of Cambridge, 2019), pp.182–3.

35. [George Vancouver], *A Voyage of Discovery to the North Pacific Ocean, and round the World*, 3 vols (London: G. G. and J. Robinson and J. Edwards, 1798), Vol. II, pp.46, 99–101, 439.

36. Anon., *A Voyage round the World, in His Majesty’s Ship the Dolphin, Commanded by the Honourable Commodore Byron* (London: J. Newbery and F. Newbery, 1767), pp.16–17. On this account see Robert E. Gallagher (ed.), *Byron’s Journal of His Circumnavigation, 1764–1766* (London and New York: Routledge, 2016), pp.lxxiii–vi.

37. Liliane Hilaire-Pérez and Catherine Verna, “Dissemination of Technical Knowledge in the Middle Ages and the Early Modern Era: New Approaches and Methodological Issues,” *Technology and Culture* 47:3 (2006): 536–65.

38. Dániel Margócsy and Mary Brazelton, “Techniques of repair, the circulation of knowledge, and environmental 3 transformation: Towards a new history of transportation,” *History of Science* 61 (2023): 3–18 and Bronwen Everill, “‘For the Services of Shipwrights, Coopers, and Grummettas’: Freetown‘s Ship Repair Cluster in Nineteenth-Century Sierra Leone,” *History of Science* 61 (2023): 60–76.

39. Hawkesworth, *Account*, Vol. I, p.7 (note 2).

40. Helen Wallis (ed.), *Carteret’s Voyage round the World 1766–1769*, 2 vols (Cambridge: Cambridge University Press, 1965), Vol. I, pp.76–93, 213–42; Vol. II, pp.365–414, 419–20.

41. Ibid., Vol. I, pp.250–4; Vol. II, pp.414–37.

42. William Robert Broughton, *A Voyage of Discovery to the North Pacific Ocean* (London: T. Cadell and W. Davies, 1804), p.2.

43. Randolph Cock, “‘The Finest Invention in the World’: The Royal Navy’s Early Trials of Copper Sheathing, 1708–1770,” *The Mariner’s Mirror* 87:4 (2001): 446–59; Crewe, *Yellow Jack*, pp.213–15 (note 8).

44. Broughton, *Voyage*, pp.9, 32, 51–4 (note 42).

45. [Vancouver], *Voyage*, Vol. I, pp.xi, xv–xvi, 4, 6, 9–10; Vol. III, p.126 (note 35).

46. Wallis, *Carteret’s Voyage*, Vol. I, pp.20–8, 105–8, 111–15, 142–4; Vol. II, p.443 (note 40).

47. Ibid., Vol. II, p.473.

48. Hawkesworth, *Account*, Vol. I, p.3 (note 2).

49. Ibid., Vol. I, p.364.

50. James Cook and James King, *A Voyage to the Pacific Ocean*, 3 vols (Dublin: E. Chamberlaine, 1784), Vol. I, p.33.

51. [Vancouver], *Voyage*, Vol. III, pp.160, 406, 409, 410, 455 (note 35).

52. Ibid., Vol. III, p.455.

53. Ibid., Vol. III, pp.455–6.

54. Matthew Flinders, *A Voyage to Terra Australis*, 2 vols and Atlas (London: G. and W. Nicol, 1814), Vol. I, p.8.

55. See e.g.: J. K. Laughton and Andrew C. F. David, “Flinders, Matthew (1774–1814),” in *ODNB* (2011) <www.oxforddnb.com/view/article/9750> [accessed 21/10/2014]; Ernest Scott, *The Life of Captain Matthew Flinders, R.N.* (Cambridge: Cambridge University Press, 2011 [1914]); Kenneth Morgan, *Matthew Flinders, Maritime Explorer of Australia* (London: Bloomsbury Academic, 2016); Kenneth Morgan, “Matthew Flinders and the Charting of Australia’s Coasts, 1798–1814,” *Terrae Incognitae* 50:2 (2018): 115–45; Eóin Phillips, “Remembering Matthew Flinders,” *Journal for Maritime Research* 14:2 (2012): 111–19; Dany Bréelle, “Matthew Flinders’s Australian Toponymy and its British Connections,” *The Journal of the Hakluyt Society* (November 2013): 1–41.

56. Flinders, *Voyage*, Vol. II, pp.135–6 (note 54).

57. Ibid., Vol. II, p.141.

58. Ibid., Vol. II, pp.141–3.

59. Ibid., Vol. II, p.143.

60. Ibid., Vol. II, pp.247–8.

61. Ibid., Vol. II, pp.274–5.

62. N. T. Geeson and R. T. Sexton, “H.M. Sloop *Investigator*,” *The Mariner’s Mirror* 56:3 (1970): 275–98, 281–3.

63. Flinders, *Voyage*, Vol. II, pp.275–9 (note 54).

64. Ibid., Vol. II, pp.348–71, 485.

65. Morgan, *Flinders, Maritime Explorer*, pp.199–200 (note 55).

66. Mark Richards, “The Matthew Flinders Memorial Statue,” <www.markrichards.eu/matthew-flinders> [accessed 22/04/2019]; “Memorial Statue,” *Flinders Memorial*, <www.flindersmemorial.org/the-matthew-flinders-memorial-statue/> [accessed 22/04/2019]. Replicas of the statue are also at Flinders University and Port Lincoln, Australia.

67. François Bellec, “La cartographie de l’Australie: compétition et coopération outre mer (XVIII^e^-XIX^e^ siècles),” *Australian Journal of French Studies* 41:2 (2004): 33–42; Thomas J. Schlereth, *The Cosmopolitan Ideal in Enlightenment Thought: Its Form and Function in the Ideas of Franklin, Hume, and Voltaire, 1694–1790* (Notre Dame, IN and London: University of Notre Dame Press, 1977), pp.25–46, 132–5; John Gascoigne, *Science in the Service of Empire: Joseph Banks, the British State and the Uses of Science in the Age of Revolution* (Cambridge: Cambridge University Press, 1998), esp. ch.6.

68. J. B. Harley, “Maps, Knowledge, and Power,” in Denis Cosgrove and Stephen Daniels (eds.), *The Iconography of Landscape: Essays in the Symbolic Representation, Design and Use of Past Environments* (Cambridge: Cambridge University Press, 1988), pp.277–312, esp. 282–3; Michel Foucault, *Power/Knowledge: Selected Interviews and Other Writings 1972–1977*, Colin Gordon (ed.), (Harlow: Longman, 1980), pp.74–5; Bréelle, “Australian Toponymy,” 17 (note 55).

69. Harley, “Maps,” pp.299–300 (note 68); Naylor and Schaffer, “Nineteenth-Century Survey Sciences,” 142–3 (note 14).

70. Harley, “Maps,” pp.279–82, 300–3 (note 68); Les Roberts, “Mapping Cultures: A Spatial Anthropology,” in Les Roberts (ed.), *Mapping Cultures: Place, Practice, Performance* (Basingstoke: Palgrave Macmillan, 2012), pp.1–25, 12–13; Rob Kitchin, Martin Dodge, and Chris Perkins, “Introductory Essay: Power and Politics of Mapping,” in Martin Dodge, Rob Kitchin, and Chris Perkins (eds.), *The Map Reader: Theories of Mapping Practice and Cartographic Representation* (Chichester: Wiley-Blackwell, 2011), pp.388–94; Foucault, *Power/Knowledge*, p.69 (note 68).

71. Flinders, *Voyage*, Atlas, Plate I, *c*.32° S, 125° E (note 54); Bréelle, “Australian Toponymy,” 13, 18 (note 55); Scott, *Life*, pp.209–14, 465–74 (note 55); on the hegemonic significance of place-naming, see Jan Tent and Helen Slatyer, “Naming Places on the ‘Southland’: European Place-Naming Practices from 1606 to 1803,” *Australian Historical Studies* 40 (2009): 5–31, 9–10, 15.

72. Royal Greenwich Observatory Archives (Board of Longitude project, University of Cambridge), RGO, 14/51, Papers on the Tides and Trade Winds, 1780s–1820s, ff.171–3, Matthew Flinders to Joseph Banks, August 23, 1804.

73. Harley, “Maps,” pp.282–3, 289–90 (note 68); Tent and Slatyer, “Naming Places,” 9 (note 71).

74. Flinders, *Voyage*, Vol. I, pp.v–vi (note 54); on Flinders’s methods see M. K. Barritt, “Matthew Flinders’s Survey Practices and Records,” *The Journal of the Hakluyt Society* (March 2014): 1–15; Morgan, “Matthew Flinders, Charting” (note 55).

75. On this concept see Roberts, “Mapping Cultures,” p.17 (note 70).

76. Sorrenson, “Ship as a Scientific Instrument,” 228–36 (note 16).

77. Measured with a ruler, the distance is 4.84 inches. The chart is on a scale of 0.5 inches to the degree of longitude. The value of longitude degrees varies with the latitude. This being a Mercator projection, however, 1’ latitude equals 1 nautical mile, so distances can be calculated reporting the value in inches onto the latitude scale. We obtain 6° 35’, which then gives 6×60+35=395 nm in three days, that is, 5.49 nm per hour (a knot equals 1 nm/h). For this method, see Mark Monmonier, *Rhumb Lines and Map Wars: A Social History of the Mercator Projection* (Chicago and London: University of Chicago Press, 2004), p.81.

78. Flinders, *Voyage*, Vol. I, p.46 (note 54).

79. See Howard I. Chapelle, *The Search for Speed under Sail 1700–1855* (London: G. Allen & Unwin, 1968), pp.25–31. Naturally, lacking the *Investigator*’s exact hourly speed, which, together with construction and waterline details, would allow us to establish its “speed-length ratio,” a shipbuilder would consider this a vague assessment, but it seems sufficient for our purposes here. For the Beaufort equivalents of eighteenth-century terminology see R. García-Herrera et al., *CLIWOC Multilingual Meteorological Dictionary, An English-Spanish-Dutch-French Dictionary of Wind Force Terms Used by Mariners from 1750–1850* (De Bilt: Koninklijk Nederlands Meteorologisch Instituut, 2003), esp. pp.14, 35.

80. Flinders, *Voyage*, Vol. II, p.271 (note 54).

81. Ibid., Vol. II, p.275.

82. Ibid., Vol. I, p.28; on Royal Navy numbers see N. A. M. Rodger, *The Command of the Ocean – A Naval History of Britain 1649–1815* (London: Allen Lane, 2004), p.608.

83. Flinders, *Voyage*, Vol. I, pp.19, 21 (note 54).

84. Howe et al., “Paradoxical Infrastructures” (note 18).

## Appendix

**Appendix.** Principal British naval journeys of discovery, 1764–1803.^85^

Voyage	Dates	Duration (in days)
John Byron – HMS *Dolphin*	3 July 1764 (Plymouth)	675
	9 May 1766 (Downs)	
Patrick Mouat / James Cumming – HMS *Tamar*	3 July 1764 (Plymouth)	750
	23 July 1766	
Samuel Wallis – HMS *Dolphin*	21 August 1766 (Plymouth)	638
	20 May 1768 (Downs)	
Philip Carteret – HMS *Swallow*	21 August 1766 (Plymouth)	942
	20 March 1769 (Spithead)	
James Cook – HMB *Endeavour*	25 August 1768 (Plymouth)	1051
	12 July 1771 (Thames)	
James Cook – HMS *Resolution*	13 July 1772 (Plymouth)	1112
	30 July 1775 (Portsmouth)	
Tobias Furneaux – HMS *Adventure*	13 July 1772 (Plymouth)	731
	14 July 1774 (Spithead)	
Constantine John Phipps – HMS *Racehorse* and HMS *Carcass*	4 June 1773 (Little Nore)	117
	29 September 1773 (Deptford)	
James Cook – HMS *Resolution*	12 July 1776 (Plymouth)	1548
	7 October 1780 (Thames)	
Charles Clerke – HMS *Discovery*	1 August 1776 (Plymouth)	1528
	7 October 1780 (Thames)	
William Bligh – HMS *Providence*	2 August 1791 (Spithead)	736
	7 August 1793 (Deptford)	
George Vancouver – HMS *Discovery*	1 April 1791 (Falmouth)	1626
	13 September 1795 (Shannon)	
William Robert Broughton / Peter Puget – HMS *Chatham*	1 April 1791 (Falmouth)	1660
	17 October 1795	
William Robert Broughton – HMS *Providence*	15 February 1795 (Plymouth)	N.A.
	DNR (wrecked)	
Matthew Flinders – HMS *Investigator*	18 July 1801 (Spithead)	N.A.
	DNR (as such)	

Some dates are in nautical time and some in civil time, but because each journey’s departure and arrival dates come from the same source the duration ought to be unaffected.

